# Spatial and temporal stability in the genetic structure of a marine crab despite a biogeographic break

**DOI:** 10.1038/s41598-022-18368-5

**Published:** 2022-08-20

**Authors:** David Veliz, Noemi Rojas-Hernández, Caren Vega-Retter, Camila Zaviezo, Ignacio Garrido, Luis Miguel Pardo

**Affiliations:** 1 Departamento de Ciencias Ecológicas, Facultad de Ciencias, Universidad de Chile, Santiago, Chile; 2 Centro de Ecología y Manejo Sustentable (ESMOI), Coquimbo, Chile; 3 Instituto de Ciencias Marinas y Limnológicas, (ICML), Laboratorio Costero de Recursos Acuáticos de Calfuco (LCRAC), Facultad de Ciencias, Universidad Austral de Chile, Valdivia, Chile; 4 Centro FONDAP de Investigación de Dinámica de Ecosistemas Marinos de Altas Latitudes (IDEAL), Valdivia, Chile; 5 Québec Océan, Département de Biologie, Université Laval, Québec, Canada

**Keywords:** Ecology, Genetics

## Abstract

Elucidating the processes responsible for maintaining the population connectivity of marine benthic species mediated by larval dispersal remains a fundamental question in marine ecology and fishery management. Understanding these processes becomes particularly important in areas with a biogeographic break and unidirectional water movement along the sides of the break. Based on variability at 4209 single-nucleotide polymorphisms in 234 individuals, we determine the genetic structure, temporal genetic stability, and gene flow among populations of the commercially important mola rock crab *Metacarcinus edwardsii* in a system in southern Chile with a biogeographic break at latitude 42°S. Specimens were collected at eight sites within its geographic distribution, with collection at four of these sites was performed twice. Using population genetic approaches, we found no evidence of geographic or temporal population differentiation. Similarly, we found no evidence of an effect on gene flow of the biogeographic break caused by the the West Wind Drift Current. Moreover, migration analyses supported gene flow among all sites but at different rates for different pairs of sites. Overall, our findings indicate that *M. edwardsii* comprises a single large population with high levels of gene flow among sites separated by over 1700 km and demonstrate temporal stability in its genetic structure.

## Introduction

Most adult benthic marine invertebrates have limited mobility or are sessile, as the larval stage is the period that enables dispersion^1^. Although the larval period only represents approximately 1–5% of the total life cycle of these species, it is important for population connectivity^2^ because it is fundamental to maintaining the cohesiveness of benthic populations, enabling them to persist through ecological and evolutionary time^3^.

Factors influencing marine population connectivity and larval dispersal include planktonic larval duration, larval behavior, and oceanographic currents^4,5^. Planktonic larval duration is positively correlated with dispersal distance^6^ and depends on the species, ranging from merely hours among corals^7^ to a year among lobsters^8^. In addition, larval behavior contributes to changes in magnitude and direction of larval movement, enabling offshore transport or residence near the coast^9^. One of the most important larval behaviors described in the water column is active vertical migration^10^. This behavior could regulate larval engagement with physical forcing and circulation, which would prevent them from straying from the coast^11^. Finally, all scales of oceanic water movement affect larval dispersal. Small-scale processes (e.g., turbulence, small eddies, and stagnant zones) combine with nearshore physical processes (e.g., waves, winds, and tides) to affect the degree of larval retention in each system^12^, while mesoscale behaviors (e.g., meanders) and global-scale physical processes (e.g., the main oceanographic currents) affect the degree of larval dispersal and population connectivity at a large scale^5,13^.

At large scales, the hydrographic regime and bottom topography affect biogeographic breaks. Examples include Cape Cod in the Atlantic Ocean^14^, Point Conception in North America^15^, and latitudes 30°S and 42°S in Chile^16^. The biogeographic break at 30°S is likely caused by differences in eddy kinetic energy (high south of and low north of 30°S) and equatorward wind (strong and variable south of and weak but persistent north of 30°S)^17^. The biogeographic break at 42°S is caused by the collision of the West Wind Drift Currents (Antarctic Circumpolar Current) with the Chilean coast, producing the northward Humboldt (Peru) Current and southward Cape Horn Current^18,19^. These biogeographic breaks act as barriers for species with low dispersal^20,21^ and promote non-random spatial movements of larvae in plankton, such as unidirectional movement^22^ or asymmetric dispersal among populations^23^. Overall, these factors can impact the survival of the planktonic larvae^24^, stochasticity in the recruitment of benthic species^25^, and the substantial changes in allelic frequencies observed when different cohorts are analyzed^26^.

Among the species whose distribution spans the 42°S biogeographic break (see Lancellotti and Vásquez (2000)^16^ for examples) is the mola rock crab *Metacarcinus edwardsii*, an important commercial fishery species that accounts for 75% of the Chilean artisanal cancrid fishery industry^27^. *M. edwardsii* typically inhabits sandy, muddy, and gravel substrates up to 70 m deep in intertidal and subtidal zones. Its distribution stretches from Guayaquil (Ecuador) to the Strait of Magellan (Chile)^28^, crossing the biogeographic breaks at 30° and 42°S.

*Metacarcinus edwardsii* attains sexual maturity at a carapace width of 100 mm for both sexes^29^. Females mate with more than one male during the breeding season, but only one male becomes the genitor of the entire egg clutch^30,31^. Hundreds of full-sib zoea are released from each female^32^. Megalopae are abundant during October and December, coinciding with the advection of warm waters towards the coast^33^. Larvae recruit in association with the estuarine environment^34^. Laboratory experiments have demonstrated a planktonic larval duration of 60 days at 15 °C^35^, and a population genetic study based on eight microsatellites found high gene flow among populations over 700 km apart^36^. However, the possible effect of the Humboldt Current on gene flow, temporal genetic stability, and population differentiation of the species across the biogeographic break at 42°S remains unknown.

In this study, we examine the spatial and temporal genetics of the *M. edwardsii* population using 4209 single nucleotide polymorphisms (SNPs). We explore the genetic differentiation, temporal stability, and gene flow patterns of *M. edwardsii* populations throughout their geographical distribution along the path of the Humboldt Current. Our study covers 1700 km of coastline, and we analyzed samples obtained in two different periods (2013–2014 and 2020–2021) on both sides of the biogeographic break at 42°S. Furthermore, we evaluate different migration models to infer the most probable gene flow patterns among *M. edwardsii* populations.

## Results

### SNP calling

From the 274 individuals collected, 20,230 raw SNPs were obtained. Following the removal of low-quality and outlier SNPs, 4209 SNPs and 234 individuals were retained for analysis. Many of the samples that failed genotyping were collected in 2013–2014, and this was likely due to poor DNA quality. The sample sizes before and after filtering are presented in Table 1.

**Table 1:** Summary of SNPs data of the crab *M. edwardsii* including sampling time (2013–2014 and 2020–2021), geographical coordinates, sample size before and after filtering, allelic richness (AR) observed heterozygosity (Hobs.), expected heterozygosity (Hexp.), expected heterozygosity corrected for sampling bias (Hn.b.) and F_IS_ at each study site.

Sites	Coordinates	N Initial	N after filtering	AR	Hobs	Hexp	Hn.b	F_IS_
2013–2014								
Tome	36° 21′ S; 72° 50′ W	23	18	1.5	0.120	0.134	0.138	0.192
Valdivia	39° 51′ S; 73° 23′ W	23	17	1.48	0.099	0.131	0.135	0.279
Ancúd	41° 50′ S; 73° 51′ W	22	9	1.41	0.084	0.124	0.132	0.380
Quellón	43° 08′ S; 73° 36′ W	22	10	1.44	0.100	0.126	0.133	0.256
2020–2021								
Valparaíso	33° 01′ S; 71° 39′ W	28	28	1.56	0.131	0.137	0.139	0.061
Dichato	36° 31′ S; 72° 57′ W	24	24	1.56	0.132	0.137	0.140	0.063
Tumbes	36° 38′ S; 73° 05′ W	10	10	1.5	0.132	0.132	0.139	0.053
Valdivia	39° 51′ S; 73° 23′ W	31	31	1.56	0.128	0.136	0.138	0.072
Ancud	41° 50′ S; 73° 51′ W	31	31	1.54	0.122	0.134	0.136	0.108
Quellón	43° 08′ S; 73° 36′ W	30	27	1.55	0.129	0.137	0.140	0.082
Aysén	45° 26′ S; 72°55′ W	30	29	1.54	0.124	0.135	0.137	0.093

### Genetic diversity and population genetic analyses

Allelic richness (AR) and expected heterozygosity (Hexp) were similar across sites and time periods. Observed heterozygosity (Hobs) varied between 0.084 in Ancúd (2013) and 0.132 in Dichato and Tumbes (2021). Higher values of *F*_IS_ (inbreeding coefficient) were observed at sites with the smallest sample sizes (Table 1).

**Figure 1:**
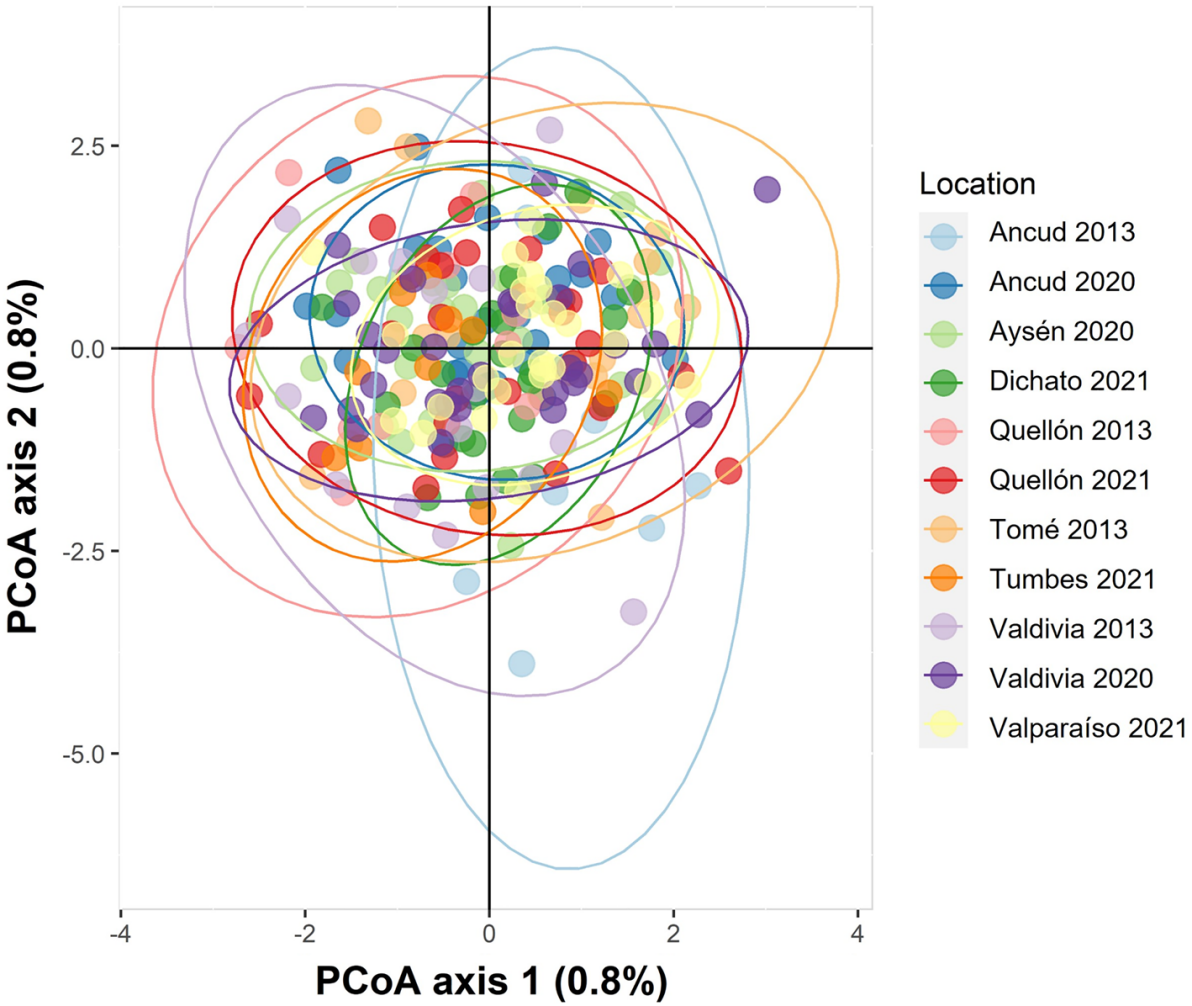
Principal coordinate analysis (PCoA) performed with *M. edwardsii*. The first and second Principal Components (x-axis and y-axis, respectively) capture 0.8% of the total variance each.

**Figure 2:**
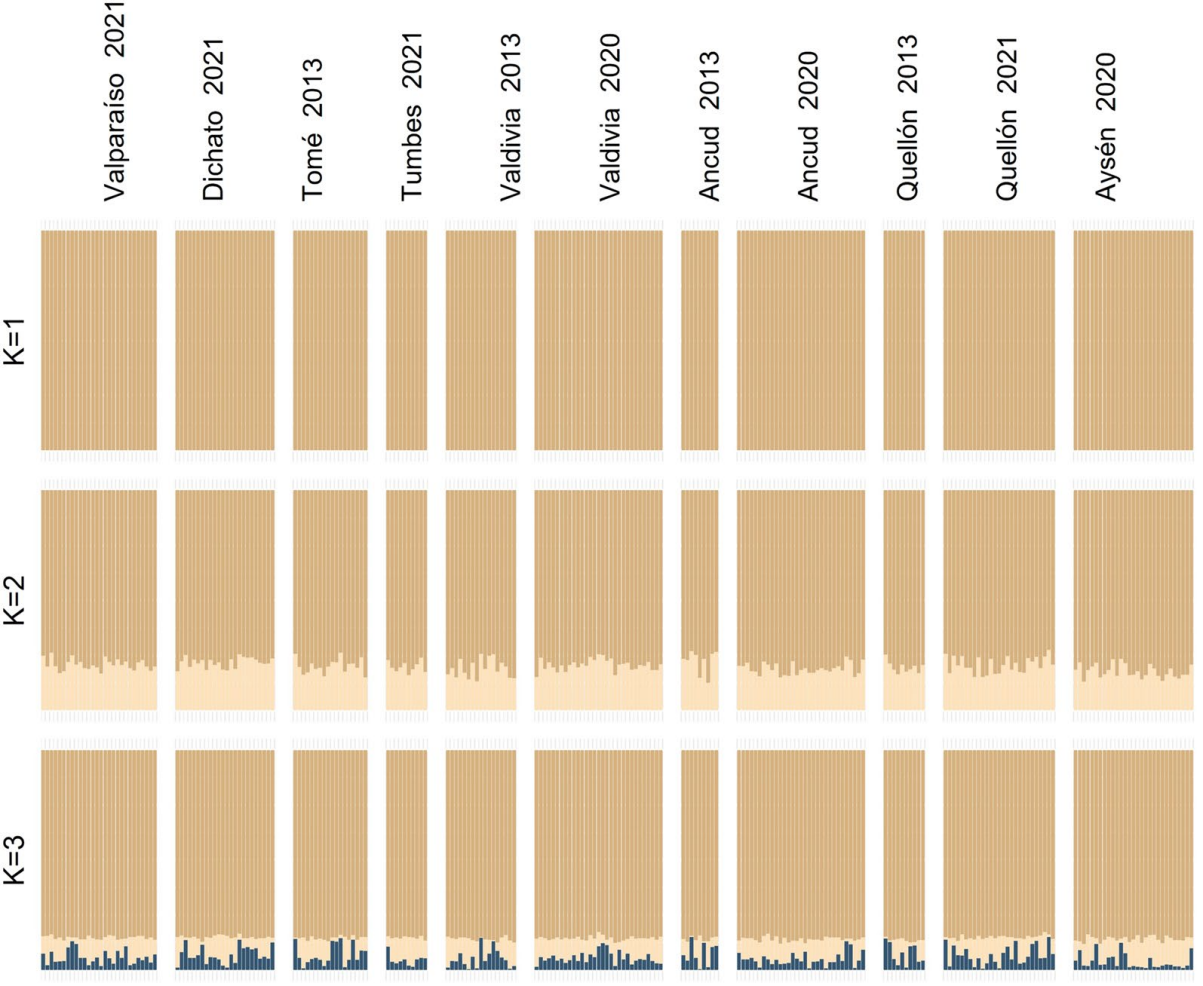
Population structure of the crab *M. edwardsii* inferred using the software STRUCTURE for *K* = 1 to *K* = 3 of the 234 individuals analyzed after filtering SNPs. A vertical bar represents each individual, and each color represents the probability of belonging to one of the K genetic clusters.

The population genetic analysis of all sites did not detect significant genetic differences among them. A principal coordinate analysis (PCoA) found individuals from all sites to overlap in the multivariate space, indicating no genetic differentiation among populations (Fig. 1). Pairwise F_ST_ values among sites were not statistically significant, except for comparisons between sites from the 2013–2014 period with small sample sizes and the other sites (Table 2). The Bayesian method implemented in STRUCTURE software found K = 1 to have the highest ln(K) value (mean LNP[K] = − 476,972.5, *P* = 0.999). Barplots of higher values of K showed no evidence of differences among sites, even from different years. Therefore, K = 1 represented the biological model with the greatest support in our dataset (Fig. 2).

**Table 2:** Pairwise FST (above diagonal) and corrected P-value (below diagonal) values for the sample sites.

	Valparaíso 2021	Dichato 2021	Tome 2014	Tumbes 2021	Valdivia 2013	Valdivia 2020	Ancud 2013	Ancud 2020	Quellón 2013	Quellón 2021	Aysén 2020
Valparaíso 2021		0.002	0.002	0.002	0.003	0.001	0.009	0.001	0.005	0.001	0.001
Dichato 2021	0.037		0.002	0.001	0.002	0.001	0.007	0.002	0.002	0.002	0.001
Tome 2014	0.051	0.057		0.000	0.000	0.002	0.005	0.001	0.001	0.002	0.002
Tumbes 2021	0.124	0.299	0.488		− 0.003	0.001	0.006	0.001	0.000	0.001	0.002
Valdivia 2013	0.018	0.051	0.493	0.976		0.001	0.002	0.001	0.001	0.003	0.003
Valdivia 2020	0.032	0.203	0.078	0.303	0.153		0.009	0.000	0.004	0.001	0.001
Ancud 2013	0.000	0.008	0.037	0.037	0.203	0.000		0.007	0.002	0.008	0.009
Ancud 2020	0.037	0.024	0.209	0.393	0.338	0.488	0.000		0.003	0.001	0.001
Quellón 2013	0.008	0.112	0.320	0.584	0.406	0.015	0.256	0.026		0.004	0.004
Quellón 2021	0.260	0.038	0.048	0.203	0.011	0.075	0.008	0.258	0.032		0.000
Aysén 2020	0.051	0.053	0.021	0.064	0.011	0.086	0.000	0.100	0.011	0.361

### Patterns of reciprocal migration among sampling sites

**Figure 3:**
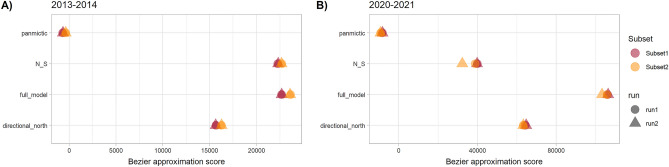
Values of the Bezier approximation score for the different models tested with MIGRATE software separately for 2013–2014 and 2020–2021. Panmictic = panmixia model; N_S = northbound migration at sites north of the biogeographic break and southbound at sites south of the biogeographic break; full model = full migration; directional_north = directional migration towards the north. The full model had the highest Bezier approximation value (*P* > 0.999) with both datasets.

**Figure 4:**
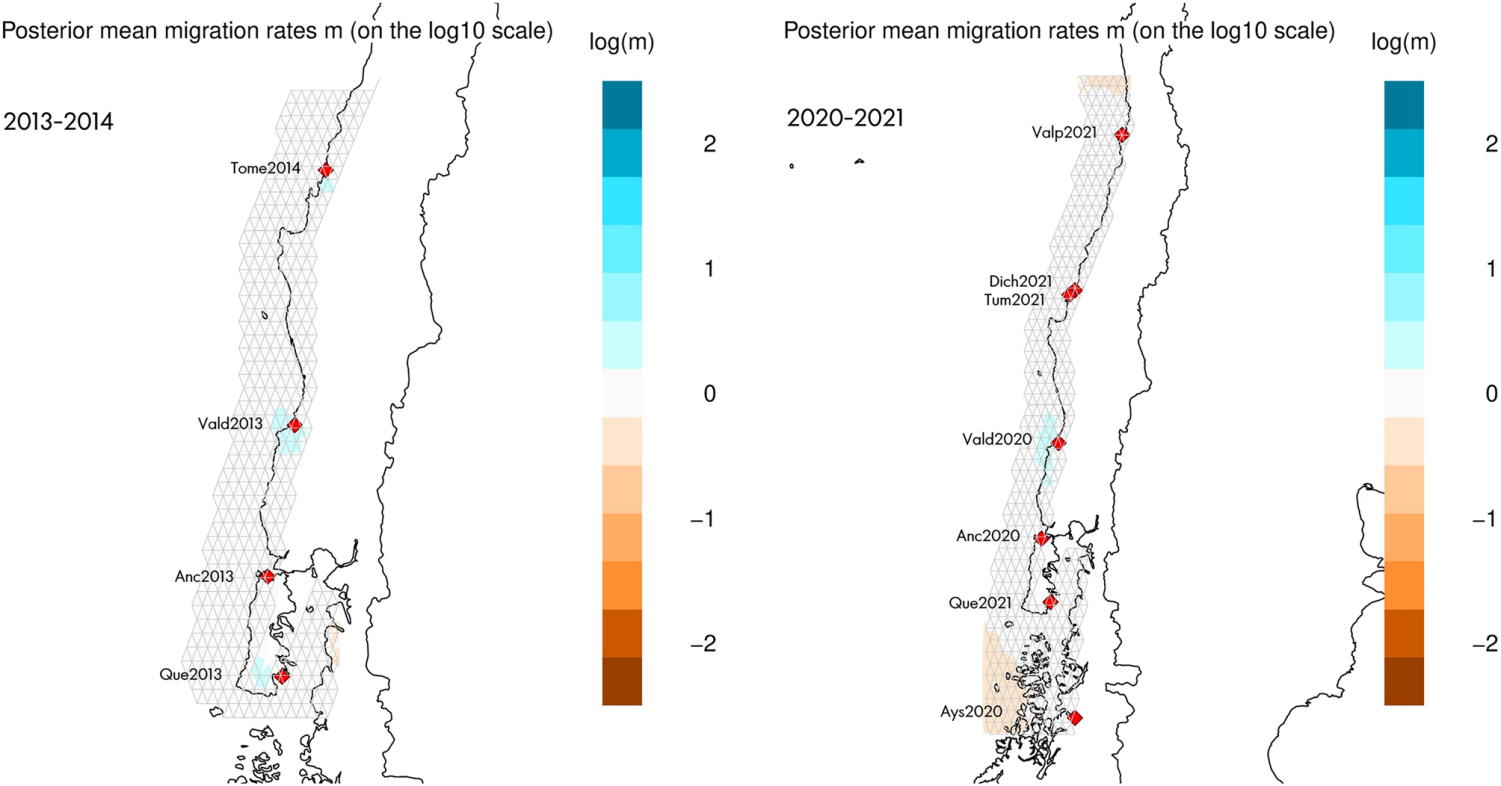
Effective Migration Rates for 2013–2014 (left) and 2020–2021 (right) estimated with the EMMS software^37^. Log(m) denotes the effective migration rate on a log_10_ scale relative to the overall migration rate throughout the habitat. The blue colors represent areas where the effective migration is higher than average, while brown colors represent areas where effective migration is lower than average. Maps drawn using library rEEMSplots^38^ implemented in R software^39^.

**Figure 5:**
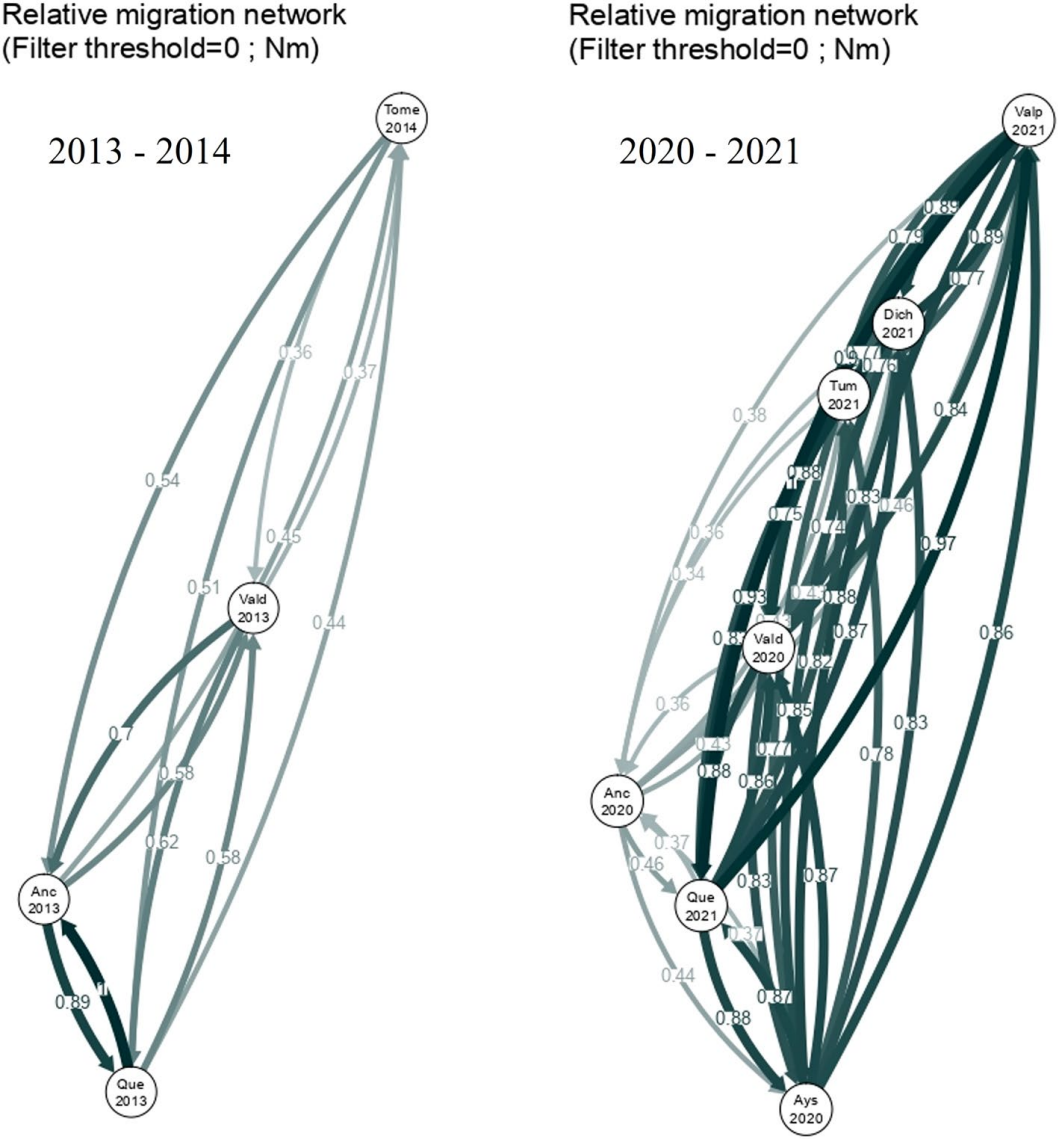
Migration network for 2013–2014 (left) and 2020–2021 (right) determined with divMigrate^40^. Circles represent sampling sites, and each arrow represents the direction and magnitude (arrow edge value) of the relative migration between sites.

Among the models evaluated with MIGRATE software, the full model had the highest Bezier approximation scores for both the 2013–2014 and 2020–2021 datasets (Fig. 3). This result indicates that gene flow occurs between all sites. Migration rate estimates obtained with EEMS had log(m) ~ 0 for all sites in the 2013–2014 and 2020–2021 datasets independently, indicating gene flow among sites (Fig. 4) and suggesting the absence of barriers to migration among sites. Finally, while a divMigrate analysis indicated strong gene flow among sites for both 2013–2014 and 2020–2021 (Fig. 5), bootstrap did not detect significant asymmetric gene flow among them (*P* > 0.05). Overall, the proportion of migrants between pairs of sites appeared to vary based on the type of analysis performed, all three methods used (MIGRATE, EEMS, and divMigrate) consistently indicated gene flow among all sites for both time periods.

## Discussion

The results of this study show a clear pattern of high gene flow among *M. edwardsii* populations and temporal genetic stability across its geographical distribution. In addition, they indicate that the biogeographic break at 42°S and the Humboldt Current do not have a significant impact on gene flow among its populations. Compared to a previous study using eight microsatellites that sampled *M. edwardsii* from 700 km of the Chilean coastline, we found similar results in a broader geographical area.

Gene flow over large geographic areas has also been reported for other crustacean species with a similar duration of planktonic larval development (two to three months), for example, the Chilean mole crab *M. edwardsii* has a planktonic larval phase of 60 days^35^, the brown crab *Cancer pagurus* of three months^41^, the Norway lobster *Nephrops norvergicus* of four to eight weeks^42^ and the blue crab *Callinectes sapidus* of five to ten weeks^43^. For these species, population genetic analyses not showed statistical differences among populations of *M. edwardsii* separated by 1700 km (this study), populations of *C. pagurus* separated by 1300 km (from the Norwegian Sea to the Kattegat straits)^44^, populations of *N. norvergicus* separated by 500 km in southern Iceland^42^ and populations of *C. sapidus* separated by 740 km in the Brazilian coast^45^.

Biogeographic breaks generally produce discontinuity between populations located on either side. Evidence for this phenomenon has been observed in different coastal areas. For example, genetic differences were observed for gobiid fishes located on opposite sides of the Mona Passage in the Caribbean Sea^46^, for different benthic invertebrates on the North and South islands of New Zealand^47^ and the Southeast Australian Biogeographic Barrier^48^, and for stomatopods located in Northern and Southern Indonesia^49^. However, the biogeographic discontinuities are not impermeable for all species. Previous studies have demonstrated that two-thirds of benthic species with pelagic development do not present a genetic discontinuity between Alaska and California^21^ and show that populations of echinoderms and some crustaceans do not show discontinuity either side of the 30°S biogeographic break in Chile^20^.

In the Chilean biogeographic break at 42°S, there is evidence of species with different propagule (zoospores or larvae) duration inhabiting both sides of the zone. The kelp species *Macrocystis pyrifera*, whose zoospores quickly settle a few meters from their parents^50^, and the mytilid *Mytilus chilensis*, whose planktonic larval phase lasts between 20 and 45 days^51,52^, show genetic differences between the two zones^50,53^. However, for the commercial gastropod *Concholepas concholepas* that have a planktonic larval duration of three months^54^, Cardenas et al.^55^ did not detect genetic differences in populations located at north and south of the 42°S biogeographic break. Therefore, as would be expected for a species with the longest period of planktonic larval development, this break was found not to affect gene flow among its populations. Overall, the analysis suggest that the duration of the planktonic larval stage is an important aspect in the gene flow of species inhabiting both sides of a biogeographic break.

An interesting issue observed in *M. edwardsii* is the temporal stability of the genetic variability across time periods (2013–2014 and 2020–2021). The analyses did not detect population differentiation, except for slight but significant differences in F_ST_ when pairs of sites with small sample sizes were compared. This temporal stability has been previously described for this species; Rojas-Hernández et al.^36^ did not detect differences in microsatellite variability in four cohorts of megalopas (over the period 2011 to 2014) collected at Los Molinos (39° 51′ S; 73° 23′ W). To our knowledge, there is little evidence of the temporal genetic stability of populations of benthic marine organisms. For example, temporal genetic stability was described in the crab *Carcinus maeneas* in the Iberian Peninsula^56,57^ and in the crab *Cancer pagurus* in Sweden^42^. Temporal genetic similarity of different adult cohorts was also studied in the Pacific Geoduck Clam *Panopea generous*^58^ and the Arctic surf clam *Mactromeris polynyma*^59^. Until now, there have been limited studies on temporal genetic stability as compared to those describing changes in allele frequencies between generations in other species of benthic marine organisms^60,61^.

It is important to note that we expected a northward asymmetric gene flow following the Humboldt Current. However, the migration analyses suggest that gene flow is not unidirectional in this area. The different models tested with the Migrate software showed gene flow among all sites but with different proportions between pairs of sites. This observation is supported by EEMS and divMigrate analyses, which also indicated a variable number of migrants between pairs of sites. In other studies, clear patterns of asymmetry produced by global circulation have previously been described for different species. In the Southern Ocean, the ocean currents produce asymmetry in population connectivity of the shrimp *Nematocarcinus lanceopes*^62^. In addition, the Benguela Current drives asymmetry in gene flow in the cosmopolitan bluefish *Pomatomus saltatrix*^63^, the Asia Northwestern Pacific Current in the brown seaweed *Sargassum fusiforme*^64^, and the North Pacific Current in the sea cucumber *Parastichopus californicus*^65^. Overall, our data suggest that the planktonic larval duration of *M. edwardsii* prevents the 42°S biogeographic break from negatively impacting in gene flow, consistent with the absence of spatial genetic differentiation in our results.

Finally, our results suggest that the duration of the planktonic larval stage is an important factor in determining the genetic structure of the species, allowing gene flow between distant populations despite the presence of a biogeographic break and currents that are primarily unidirectional. The findings of this study will be important for fishery management of *M. edwardsii* since artisanal fishing is partly dependent on this species in the south-central zone of Chile.

## Materials and methods

### Sampling sites

**Figure 6:**
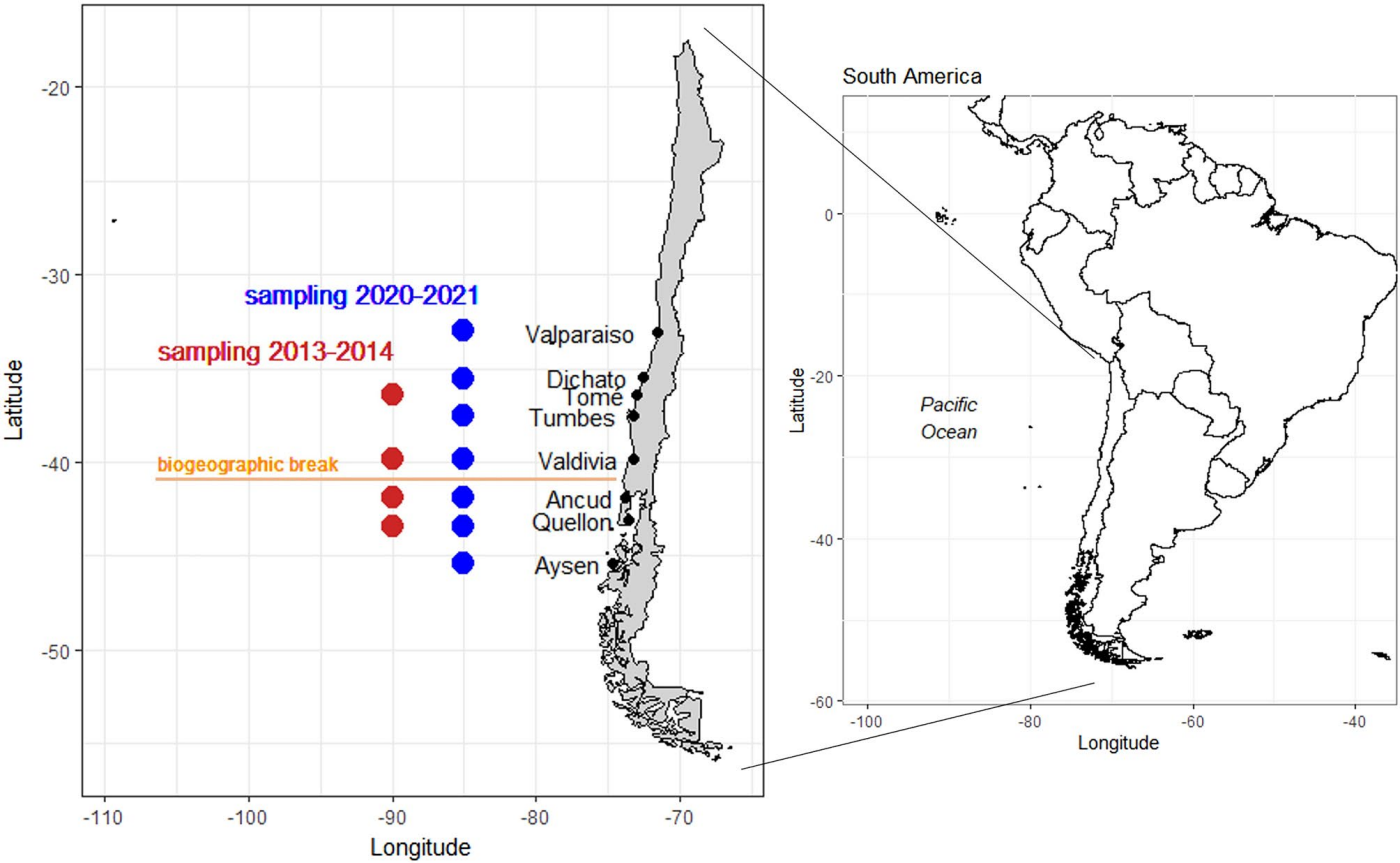
Sampling sites of *M. edwardsii* in Chile. Red points represent samples obtained in 2013–2014, while blue points represent samples obtained in 2020–2021. The horizontal line in orange represents the biogeographic break at 42°S. Maps drawn using library maps^66^ and mapdata^67^implemented in R software^68^.

A total of 274 adult *M. edwardsii* crabs were collected for this study. Nations (1975)^28^ described the geographical distribution of *M. edwardsii* from Guayaquil (Ecuador) to the Strait of Magellan (Chile). We searched for this species at different sites in its described geographic distribution in Chile however it was not found north of Valparaiso (32°S). Our sampling effort north of Valparaiso consisted of field work in Arica (18° 48′ S), Pisagua (19° 35′ S), Iquique (20° 12′ S), Antofagasta (23° 65′ S), Bahía Inglesa (27° 07′ S), and Coquimbo (29°59’S) in Chile. Our observations highlight the need for current, up-to-date biogeographical distribution for this and other crab species. We used two group of samples, first group collected in 2013–2014 and a second group collected in 2020–2021. The samples from 2013–2014 were reported by Rojas-Hernández et al.^36^ that performed a population genetic analysis using eight microsatellites and covering sites along 700 km of the Chilean coast. The samples used here and reported in Rojas-Hernández et al.^36^ were from Tomé (n = 23), Valdivia (n = 23), Ancud (n = 22), and Quellón (n = 22). The samples collected in 2020–2021 were collected in Valparaíso (n = 28), Dichato (n = 24), Tumbes (n = 10), Valdivia (n = 31), Ancud (n = 31), Quellón (n = 30), and Aysén (n = 30) (Fig. 6). Crabs were collected by local fishermen using commercial crab traps, and a pereiopod from each specimen was stored in 95% ethanol until analysis.

### Sequencing and SNP calling

For each crab, DNA was extracted from a small piece of muscle and subjected to massively parallel sequencing at Dart Diversity Arrays Technology Pty Ltd. (DArT; Canberra, Australia). Following the methods of Kilian et al.^39^, each DNA sample was digested using the restriction enzymes *PstI* and *HpaII*, and fragments > 200 bp were ligated with an 8 bp barcode prior to polymerase chain reaction (PCR) amplification. The PCR products were then standardized and sequenced using the Illumina HiSeq 2500 platform (San Diego, USA).

Sequences generated from each lane were processed using proprietary DArT PL analytical pipelines. Barcode region and complete reads were filtered by quality parameters (barcode region: Phred > 30; complete read: Phred > 10). Using DArT PL proprietary algorithm, identical sequences were collapsed into “fastcoll files” and low-quality bases from singleton tags were corrected into correct bases using collapsed tags with multiple members as a template. The proprietary DArT PL algorithm (named DArTsoft14) was used to call the SNPs. For this study, call quality was assured by a high average read depth per locus (average of all markers was more than 10 reads/locus) and a sequencing volume per sample of 1.64 million reads. A total of 83 technical replicates of the 275 unique samples were run to estimate the reproducibility of the reported markers (> 99%). All this information was provided by DArT PL. The resulting SNP data was filtered using the dartR library^69^ in the R statistical software^68^, retaining: (a) only one SNP in reads containing two or more SNPs, (b) SNPs with a read depth > 5 or < 100, and (c) SNPs with a > 95% reproducibility score, an index provided by DArT PL that indicates the proportion of replicate technical samples with a consistent marker score (reproducible result). SNPs that were monomorphic, had > 15% missing data, or a minimum allele frequency (MAF) < 1% were removed, as were samples with > 15% missing data. Based upon a relatedness value r > 0.4, we detected a pair of related individuals collected in Concepción in 2014. One individual was removed to avoid potential bias in the population genetic estimation.

All SNPs showing signs of selection were removed to avoid potential bias in estimating the differences among sampling sites. Three different approaches were used here: (a) the likelihood-based method implemented by the *outflank* function of the dartR library in R, (b) the Bayesian method implemented in the BayeScan software^70^, and (c) the relationship between F_ST_ and heterozygosity implemented in the Fsthet library^71^ in R software. We removed all SNPs that showed evidence of selection in any of these three approaches. Finally, SNPs with significant departures from Hardy–Weinberg equilibrium for one or more sites were removed using the dartR library in R, and SNPs with linkage disequilibrium > 0.2 in all sampling sites were removed using the PLINK 2.0 software^40^.

### Genetic diversity and population genetic structure

Genetic diversity at each sampling site was described with expected heterozygosity (Hexp), expected heterozygosity corrected for sampling bias (Hn.b), observed heterozygosity (Hobs), and the inbreeding coefficient (F_IS_) estimated using the GENETIX v 4.05 software^72^. Allelic richness (AR) was estimated using the divBasic function of the diveRsity library in R^72^.

Population genetic structure was estimated using the SNP database after removing all SNPs with signals of selection. Three population genetic methods were used with all data obtained in 2013–2014 and 2020–2021: (a) PCoA to describe the distribution of individuals in multivariate space, using the dartR library in R, (b) pairwise *F*_ST_ calculated using GENETIX^73^ with 5000 permutations followed by the Bonferroni correction to the P-value, and (c) estimation of the most probable number of genetic clusters (*K*) using the Bayesian approach implemented in the STRUCTURE software^74^. The admixture model and correlation of the allele frequencies were used as input. The procedure was performed three times for each *K* between 1 and 6 with a burn-in of 100,000 iterations and an after-burn-in of 200,000 iterations. The probability of each value of K was estimated as described in the STRUCTURE manual^75^.

### Patterns of reciprocal migration among sampling sites

Separately analyzing samples from periods 2013–2014 and 2020–2021, we estimated the direction and magnitude of the gene flow among sampling sites using three methods.

First, the historical migration rates was assessed using the Migrate software^76^ and two random subsets of 1000 SNPs per period. This approach employs a coalescent method to estimate mutation-scaled migration rates (M) for each group over the last 4N_E_ generations. The Bayesian inference of the Migrate software was used with the default settings except for the following run options: (a) one single long run utilizing heating with temperatures of 1.0, 1.5, 3.0, and 1,000,000; (b) 1,000,000 genealogies were run with a sample increment of 10; and (c) the first 100,000 genealogies. The uniform prior distribution was used for Θ (from 0 to 0.1) and *M* (from 0 to 100,000). We tested four models that represent the probable gene flow in the studied area: (a) panmixia model, (b) full migration, (c) directional migration towards the north (considering northern variation in ocean circulation^37^), and (d) northbound migration at sites north of the biogeographic break and southbound at sites south of the biogeographic break. To identify the best model, each analysis was performed twice for two independent subsets (a total of four runs per model), and we used the *bf.py* Python script provided by Beerli et al.^38^ that compares the Bezier log marginal likelihood values obtained in each model. MIGRATE was run at the Cyber Infrastructure for Phylogenetic Research (CIPRES, www.phylo.org).

Second, the Estimating Effective Migration Surfaces (EEMS) software was used to visualize the gene flow patterns among sampling sites. EEMS estimates migration rates so that the genetic differences observed in the data match the genetic differences expected under an idealized stepping-stone model. These estimates are then interpolated across sampling sites to produce an “estimated effective migration surface”—a visual representation of genetic variation—that highlights regions with higher-than-average and lower-than-average historical gene flow^77^. To capture the continuous population structure, *EEMS* covers the habitat with a dense regular grid, in which each deme exchanges migrants with its neighbors. If habitable regions are unsampled, estimates are based on the prior, which assumes no heterogeneity in migration rates^77^. EEMS was run with the full set of 4209 SNPs using 500 demes and three independent chains of 5,000,000 MCMC iterations with a burn-in of 1,000,000 and sampling performed every 9999 iterations. The proposed variances were adjusted considering an acceptance rate ranging from 10 to 40%. Results were plotted using the rEEMSplots package^77^ in R. Note that the results for effective migration rates are on a log_10_ scale (denoted as log(m) in the plot) relative to the overall migration rate in the habitat. Thus, a log(m) = 1 represents an effective migration ten times greater than the average, and a log(m) = -1 corresponds to an effective migration ten times less than the average.

Finally, the direction and magnitude of the gene flow between pairs of sampling sites of *M. edwardsii* were estimated using the divMigrate function of the diveRsity library^72^ in R. The Alcala’s statistic (Nm_Alcala_) was used as a distance measurement, which incorporates information from both Gst and D and maybe generally better suited to different demographic scenarios^72^. Furthermore, the asymmetry of gene flow between pairs of sites was tested with the full set of 4209 SNPs using a bootstrap of 1000 iterations with Nm_Alcala_ used as a distance measure.

## Data Availability

The datasets generated and analysed during the current study are available in the datos.uchile.cl repository, https://doi.org/10.34691/FK2/VF1R0K. Further, raw data is available in GenBank with the following NCBI data accession: BioProject ID PRJNA863944 and BioSample accessions: SAMN30070490—SAMN30070763.
